# Public Compliance Matters in Evidence-Based Public Health Policy: Evidence from Evaluating Social Distancing in the First Wave of COVID-19

**DOI:** 10.3390/ijerph19074033

**Published:** 2022-03-29

**Authors:** Caixia Wang, Huijie Li

**Affiliations:** 1 Qu Qiubai School of Government, Changzhou University, Changzhou 213159, China; caixia5w@163.com; 2 School of Public Administration, Jilin University, Changchun 130012, China

**Keywords:** COVID-19, public health crisis, social distancing policy, gathering restrictions

## Abstract

When the unprecedented COVID-19 pandemic first spread, governments could implement a wide range of measures to tackle the outbreaks. Conventional wisdom holds that public health policy should be made on the basis of empirical demonstrations, while little research has probed on how to safeguard the expected policy utility in the case of evidence shortage on novel contagious diseases. In particular, the fight against COVID-19 cannot succeed without public compliance as well as the support of people who have not tested positive. Based on the data from the first wave of COVID-19, by using a random effect estimator, fixed effect method, and hierarchical technique, we specified the efficiency of particular social distancing policies by contextualizing multiple factors. We found that adopting gathering restrictions decreased new case growth but were conditional on its interaction with population density, while mitigation effects constantly corresponded to policy magnitude in a given time; for which the effective patterns varied from three days to sixty days. Overall, policies encouraging social distancing exerted a positive effect on mitigating the first wave of COVID-19. Both the enforcing duration and public compliance constrained the expected impact of nonpharmaceutical intervention according to degrees of policy level. These findings suggest that, when evidence is incomplete, the effectiveness of public health crisis management depends on the combination of policy appropriateness and, accordingly, public compliance.

## 1. Introduction

As of March 5 of 2022, the WHO reported that there have been 440,807,756 confirmed cases of COVID-19, including nearly 6 million deaths [1]. COVID-19 plunged the world economy into the worst recession since World War II, “likely to leave long-lasting scars” [2]. In 2020, the global economy shrank by around three percent [3]. The public also suffered from disproportionate marginalization due to structural inequality [4]. Survival, freedom and other values may erode the willingness of the public to comply with restriction measures, not to mention the long-lasting social life disturbances of each person after the repeated resurgence of COVID-19 outbreaks. Nevertheless, re-opening the economy cannot resume soon without everyone’s support during the emergency responses. Furthermore, other problems are the lack of evidence on the effectiveness of gathering restrictions and by which scale is individualism justified when faced with social distancing measures [5]. In such situations, the public health policies related to fighting COVID-19 struggle to achieve complete compliance.

Few other infectious diseases have attracted so much attention from people who are not confirmed positive. To tackle the pandemic, to avoid becoming part of the transmission chain, collective endeavors from the public mattered more than ever, and yet, public engagement was regarded to be insufficient [6,7]. The virus mutated unexpectedly, and restriction measures were often lifted and imposed repeatedly [8]. Hence, doubts arose concerning the value of behavioral changes in the public. Given the only partly compelling evidence, factors decreasing adherence became emerging concerns [5,9]. To join the debate on the gap between the ideal effect and actual efficacy of lockdown measures, this article deepened research on the topic by integrating policy formulation and implementation within an interacting process. Our investigation contributed to the emerging concern on redesigning public health policy with incomplete evidence, under the assumption that public compliance was conditional in its practice, and accordingly, efficiency. Following Alfano and Ercolano (2020), this study adopted a cross-country panel analysis to estimate the efficacy of social distancing policy on the daily number of new cases [10].

COVID-19 does pose catastrophic challenges to public policy [11]. However, how should it be addressed through policy interventions when the public is important for implementation efficacy? Less light has been shed on the way to adjust social distancing policies to the right degree at the right time. If we go back to the first wave period, the puzzle of designing public health policy with incomplete evidence due to the uncertain public compliance conditions is more difficult to solve. Additionally, a retrospective study of the first wave period is therefore useful in preparing for future novel epidemics. Nevertheless, our study does not evaluate the heterogeneous performance of governmental actions in any sense. This study aims to provide some hints for the above-mentioned policy puzzle by learning comprehensively from the global policy response experience in combating COVID-19. Therefore, our research provides a general view on the collective efficacy of the self-centered national response worldwide. This work may benefit the WHO by strengthening its role in coordinating multiple shareholders’ efforts to address COVID-19.

## 2. Literature Review

### 2.1. The Differentiating Function of Evidence in COVID-19 from Traditional Public Policy

Evidence facilitated decision-makers to make public policy [12]. In the sphere of public health, covering tobacco control, chronic disease prevention, smoking bans in public areas, etc., evidence were in the forms of peer-reviewed journals, program evaluations, participant narratives and group interviews. Moreover, local data and TV news have also shaped public health policy [13,14]. The application of evidence was attributable to organizational frameworks, the availability of evidence, networking between researchers and policymakers, and the context of the country [15]. In the cognitive conversion of knowledge into policy, McCaughey and Bruning (2010) asserted that decision-makers took on a nexus role [16]. Arcand (2013) assumed that different types of policymakers statistically competed in the evidence generation [17]. In the process, both personal contact and timely relevance were closely related to the evidence utilization, and apart from that, emotions and beliefs also influenced the selection preference of decision-makers [18,19]. In the case of using incomplete evidence, advocacy played a convincing part [20].

The function of evidence varies between types of public health policy [21]. Evidence from epidemiology and evaluation benefited policy and practice [22]. Its application required the practitioners to use it in appropriate policy environments [23]. Along with that, accessible evidence was adopted into context-specific interventions [24]. However, COVID-19 is not fit for the traditional classification of public health events, as its severity resulted in a unique environment of public policy [25]. In the first wave, scientific advice provided evidence for governments [26]. Curbing the spread of the virus proved to be the goal of confinement [27]. Though the effective application of nonpharmaceutical interventions was evidenced to reduce the virus transmission, governments’ crisis capacities have been mixed in imposing these types of policy interventions [28,29]. On this emergent occasion, emerging evidence suggested that the public follow social distancing policy thanks to the finding that the virus mainly spread between people who got into physical closeness [30]. Research inferences proved that large group settings were associated with a higher probability of transmission and that a reduction in mobility led to diminishing the infection rate [31].

### 2.2. The Exploring Debate on the Determinants of Social Distancing Practice and Its Impact

Social distancing policy played a substantial role in the response to COVID-19 [32]. It was assumed to mitigate the spread of the virus, but debate emerged due to selecting the measurement of its impact [33,34]. Apart from using the COVID-19 case growth rate and mortality growth rate, Greenstone and Nigam (2020) sought empirical proof by considering deaths as a proxy of social distancing performance [35]. To evaluate it through the lens of healthcare capacity enhancement, Matrajt and Leung (2020) demonstrated that social distancing policy, coupled with testing and contact tracing, was effective in tackling the re-emergence of COVID-19 [36]. Differences in the actual efficacy appeared across particular measures [37]. For example, a mask mandate was of stronger efficiency than that of other policy instruments, such as gathering bans [38]. In that regard, some scholars found that gathering ban measures have been far more prevalent in COVID-19 than in former pandemics, but there has been insufficient concern about their specific role on their own [39].

The debate was broadened on the determinants of public compliance. Evidence has traditionally constructed the basis of public policy, while, in the first wave of COVID-19, ethnic explanations were preferred due to the shortage of evidence [40,41]. Some studies preferred probing the individual practical factors [42]. They used individualism to explain the probability of disobedience [43]. Less adherence to the guidance was related to the acceptance of anecdote theory [44], whereas factors affecting the intent to obey included perceived susceptibility and benefits [45,46]. Given that, individual morality was highlighted in public health campaigns [47]. On the basis of previous research, emerging literature focused on the conditional role of collective compliance to examine social distancing efficacy. Though individual efforts decreased the death toll, enforceable measures saved twice that number [48]. Based on that, one issue was how to design public policy in order to appropriately motivate collective efforts. Clements (2020) argued that knowledge growth was positively associated with the odds of public compliance with gathering bans [49]. However, the positive correlation between top-down restrictive measures and the percentage of public compliance, is awaited to be proven [50]. Globally, social distancing policy in its totality ran into difficulties with public compliance, considering the high levels of urbanization, social norms and the annual hosting of religious mass gatherings [51]. National characteristics limited its efficacy through party politics, the information market and social composition [52]. Moreover, it was the compliant proportion of infected individuals with mild symptoms that were directly related to the effectiveness of social distancing policies [53]. Though close contact was viewed as the main transmission route, “the certainty of becoming infected is still unpredictable” [54]. Given the discussions above, the characteristics of public policy and public compliance restrained the efficacy of COVID-19 [55]. Therefore, the social distancing policy called for implementation in a rationally layered way [56]. Following these insights, the question we address is: Under what conditions, from both policy and public perspectives, can social distancing policy exert reasonable efficacy?

## 3. Data and Methods

### 3.1. Data and Variables

In this section, we used two-panel datasets to test the conditional effect of public compliance on the efficacy of social distancing policy. A total of 17,812 observations were included from 1 January to 10 June 2020, covering 165 countries and territories. One data panel included the introduction of social distancing policy as a categorical variable, capturing whether a country or territory implemented the restrictive measures or not, and this variable was denoted by ‘gre’. The other data panel measured the magnitude of social distancing policy, and we used ‘resg’ to label it. As was shown in Table 1 and explained in the data availability statement, we mainly matched three datasets, covering the COVID-19 Coronavirus data (ECDC), the Oxford COVID-19 Government Response Tracker (OxCGRT) data and the population density data from World Bank. On the basis of the OxCGRT data, the second-panel data was formulated by selecting the four types of restrictions on gatherings, and there were 12,024 observations. In these two datasets, the dependent variable was the daily new confirmed cases. The COVID-19 Coronavirus data, collected by the epidemic intelligence team from the European Centre for Disease Prevention and Control, came from the European Union’s Open Data Portal. We employed this data for taking countries and territories as one whole unit, which allowed for matching with other covariates at the country level. Considering the exponential feature in COVID-19 dispersion, we used the logarithm of daily new cases as the dependent variable in the section of fixed effect analysis.

In the following, the variables of interest are social distancing policy and public compliance, so we used two explanatory variables, restriction on gatherings and population density, respectively, to represent them. Such an integrated logic follows the established argument that gathering prohibition leads to a reduction in human mobility by treating the public as a whole, and its mitigating efficacy may receive full evidence, assuming complete public compliance [57,58]. Regarding the variable of gathering restriction, the data were obtained from the Oxford COVID-19 Government Response Tracker (OxCGRT) [59]. In this dataset, restrictions on gatherings refer to the limits on private gatherings. The data on gathering restrictions were unique in that they adopted a uniform standard in response to subcategorization, which enabled us to compare different types of gathering bans with a choice of time setting. Moreover, we recorded a time variable based on the OxCGRT data. This acted as a timing dimension of restriction policy design. Additionally, noticing the dynamic COVID-19 transmission situation, we computed the cumulative duration of the specific restricting ban, starting from the day that the policy came into force to its termination, in the first wave of COVID-19. This novel variable was designed to test how long the specific degree of restrictive measures impacted on its differentiating efficacy.

In terms of measuring the influence of public compliance on public policy efficacy, we chose the standard population density obtained from the World Development Indicators of the World Bank in 2018. Differently from a randomized experiment or a social survey in certain local areas, we measured its significance by its link with social distancing policies. This initiative gained support from an observational study in the fieldwork [60]. In the summer of 2020, some European countries loosened their policies, which allowed people to travel inside the continent. We conducted a study in some European cities, including Paris, Copenhagen, Hamburg, Roma, Athens and Zurich. A connection between policy instructions and public compliance has been identified from this comparative analysis, and less public compliance was linked with a lack of precise mitigating measures or necessary communications in densely populated areas. As is posited in the lockdown impact literature, the situation was similar among the states of the US [61]. Given this first-hand fact, we hypothesized that public compliance varied partly according to policy characteristics. Population density has to be used as a proxy variable to measure its influence on the policy efficacy, due to the data collection limitation of actual resident behaviors in worldwide countries, in the first wave of COVID-19. Taking the evidence of rural Italy as an example, becoming infected when in a high population density was highly likely due to social proximity [62]. This finding encouraged our research to estimate the association between public compliance and social distancing, by using population density. Thus, we included the interaction term between the gathering restriction policy and population density in the model set.

### 3.2. Estimation Technique

This paper aimed to evaluate the general and evolving mitigation effect of social distancing policy in the first wave of COVID-19. The data were of unbalanced time-series and cross-sectional structure, providing the possibility to undertake random effect and fixed effect speculation in an aggregated analysis. The main results were expected to capture the dynamic partiality of social distancing policy impact. The analysis was conducted using R version 4.1.0, which is developed by the R core team and funded by the R Foundation for Statistical Computing in Vienna, Austria. Some steps were taken in this specification. First, we used the random effect model to estimate the cross-national effect of adopting social distancing policy. For this purpose, the presence of a social distancing policy included four types of gathering restrictions. To account for endogeneity concerns and to mitigate omitted-variable bias, we replicated the original regression, controlling for the randomness of continent and time (in days), and outlined the results in the Supplementary Materials. This robustness check provided consistent results in broadened geographical contexts. Technically, restriction on gatherings is a categorical variable. If we treated it as a time-invariant variable, the fixed effect examination would eliminate its effect. This consideration justifies selecting a random effect to evaluate the mitigation of general restrictions. Second, we used the LSDV estimator to examine the effects of specific measures across countries and territories. One might be concerned that restrictions on gatherings had varying contents as countries gradually lifted restrictions; to address this, we took the whole policy cycle into account. Third, our constructed dataset had a nested structure. To check the policy efficacy across more broad geographical coverage, we employed the hierarchical model as a robustness check. The result, presented in the Supplementary Materials, is in line with our argument.

## 4. Results

### 4.1. Public Compliance Conditions

We posited that public compliance exerted a conditional effect on the policy response efficiency in combating COVID-19. To test such an argument, we conducted the analysis using the interaction of social distancing policy with public compliance. Table 2 reports our main results on the random estimate of introducing gathering restrictions. We reported the results in the 1–3 column. We began analyzing the uncontextualized efficacy of adopting a gathering ban in Model 1. As was expected, the presence of applying restrictive measures associated positively with the existence of daily increasing COVID-19 cases. On average, countries and territories that adopted gathering restrictions reported approximately 938 more new cases than those that took no such restrictions when holding other explanatory variables as constant. This finding is in concordance with common sense. Geographical areas with mass COVID-19 outbreaks had a tendency to take more restrictive measures. Then, we tested whether there was a single effect of population density on social distancing efficacy, as was shown in Model 2, and it turned out to be nonsignificant.

Based on the results in Model 1 and Model 2, one may wonder whether the interaction of social distancing and population density has an impact on the COVID-19 spread. This concern resonates with our regression equation in the model part of the Supplementary Materials, and Model 3 reports the answer. The combination of a restriction on gatherings and public compliance presents a significantly decreasing effect on new cases, at the 1% level. One unit change in the interaction between population density and restriction on gatherings led to a 13.5% decrease in daily new cases, with a standard error of 0.03. It indicates that the aggregated mitigating power of social distancing relies on the support of global public compliance. This result may deny the “absolute” impact of introducing a social distancing policy on the COVID-19 spread, irrespective of policy implementation conditions, such as public compliance. Overall, public compliance conditionally affected social distancing efficacy in the first wave of COVID-19, as evidenced by the random analysis.

### 4.2. Policy Magnitude Co-Weights

To what extent is the constraining measure appropriate? Table 3 presents results on the fixed effect of specific restrictions on gathering. As is presented in Model 3, the single efficacy of social distancing policy varies with policy magnitude. Level 2 captures the most effective power in decreasing new cases and has a coefficient of about −1147 than that of the remaining levels when holding other variables as constant. Unexpectedly, Level 4 shows a nonsignificant effect. Therefore, the claim of ‘no strictest measure, no best efficacy’ finds no grounds here. After including the variable of population density into Model 2, Level 2 has a rough impact of decreasing 473 new cases, while the coefficient of Level 3 is around −404. This result means that under the same conditions, geographical areas that restrict a mass gathering of 11–100 people face 69 more new cases than locations that implement bans on gatherings of 101–1000 people on average. It infers that on specific occasions, the less strict type of restrictions has a stronger effect in terms of COVID-19 mitigation.

One may ask, will the interaction effect between social distancing and public compliance still hold irrespective of policy magnitude? We tested this puzzle by applying the same equation as that examined in Model 3 in Table 2. As is depicted in Table 3, taking Level 1 as the reference, the remaining restriction types show stronger negative effects according to the increasingly tightened policy. The interaction effect of Level 4 and population density has a coefficient of −3.898 on new more cases, marginally greater than those of Level 2 and Level 3. This statistical output shows that the strictest measure occupies a relatively stronger conditional mitigating influence than the remaining ones. Stricter restrictions have slight advantages in curbing the virus dispersion when considering public compliance. This finding indicates that the idealistic efficacy of the strictest measure is conditional on its implementation with full public compliance.

### 4.3. Policy Duration Matters

**Figure 1:**
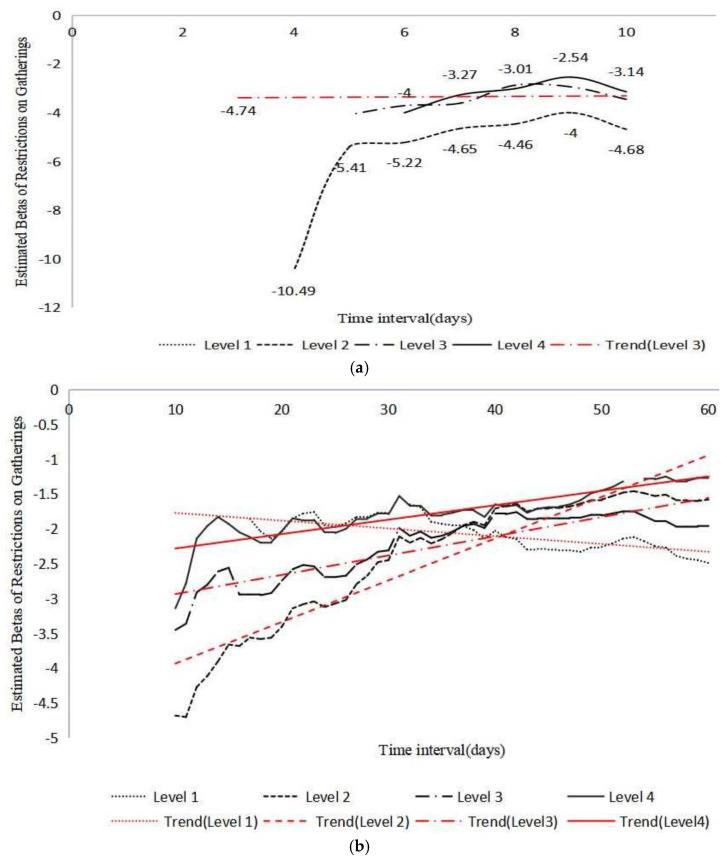
Mitigation of restriction on gatherings within 60 days. (**a**) Efficacy of restrictions on gatherings within 10 days. (**b**) Efficacy of restrictions on gatherings over time (10-60 days). Note: Betas are estimated using the least squares dummy variable (LSDV) model, the dependent variable is transformed by log, the function contains country and days (factored respectively), “resg”, the interaction between “resg” and “dnst”, and 0. The standard errors are presented in the Supplementary Materials. In (**a**), Level 1 and Level 4 are labeled with numbers, Level 1 appears to be none, interestingly. All the “Trend” lines (red color) in (**a**,**b**) are estimated with the linear model. The computation of days is included in the Supplementary Materials. All timing points have their specific contexts, tailored to the evolution pattern of COVID-19 in each country (and territory).

How long would the constraining measures be sufficient? Containment policy was predicted to have the largest gains rapidly, and an earlier response is thought to be more effective [63]. According to previous wisdom, differences lied in specific measures at certain time points [64]. Based on reviewing the extant literature, we argue that the impact of specific social distancing policy varies with its time duration, and such policy response is assumed to have an optimal timing in combatting virus transmission. Table 4 and Figure 1 illustrate the results. Table 4 reports the estimated results of gathering restrictions with a time axis from three days to sixty days. Based on the estimates, restrictions on gatherings of between 11 and 100 people (Level 3) and restrictions on gatherings of no more than 10 people (Level 4) begin to play a statistically significant mitigating role within three days, at the five-percent level, and the looser restriction rules have a more influential effect. Within seven days, a restriction on gatherings of between 101 and 1000 people (Level 2) has a negative correlation with more new cases with the coefficient of −4.646. It obtains the strongest effect with the most powerful sign, at the one-percent level. This result shows that in the same time framework, countries that ban the gathering of between 101 and 1000 people (Level 2) have about 5 cases per day fewer than countries that have no restrictions or take other types of gathering prohibition actions.

Having a detailed examination of Table 4, an evolvingly accumulative trend of social distancing efficacy is depicted by the results. In general, most gathering restrictions have optimal effects within seven days, and the correlation between the mitigation strength and restriction magnitude varies with the interval option. In Figure 1, within three days, the stricter restriction has a stronger mitigation effect, whereas, within seven days, less strict policy proves to exert a greater impact than stricter ones. Adjusting the time axis to thirty days, the effect of restriction on gatherings of more than 1000 people (Level 1) earns statistical significance, while the gathering restriction of between 101 and 1000 people (Level 2) retain its predominance over other types of restriction. Within forty days, Level 1 obtains the greatest power, and the effect of restrictions on gatherings of between 11 and 100 people (Level 3) ranks as the second, with a greater effect than those of Level 2 and Level 4. In line with previous discussions, such a hierarchical impact structure lasts until the sixtieth day. This result implies a changeable relationship between restriction magnitude and mitigation effect with a longer time horizon.

To summarize, our main findings have been further confirmed in Figure 1. Combined with short-time and longer-time frameworks, the patterns of influential restriction type partly change following the aggregate prediction. In Figure 1b, the lightest restriction (Level 1) gradually increases mitigating influence, and yet, the strictest one (Level 4), experiences a decline with an initial rapid and then slow pattern. The situations of other types are different in the short-time horizon. As is presented in Figure 1a, Level 2 and Level 3 gain explanatory power between 3 and 7 days and commonly decrease later. Taking Figure 1a,b altogether, Level 2 captures a higher efficacy in mitigating COVID-19 than that of Level 4, and this trend continues when the duration comes to more than fifty days. Overall, these variations consolidate our argument in adjusting the degree of public health policy according to its timing performance. This suggestion may work better if granted due consideration to public compliance.

## 5. Discussion

Our explorative study complemented evidence on the importance of public compliance with restriction policies, considering uncertain public compliance conditions and incomplete agreement of its necessity could drag everyone into the COVID-19 transmission chain. We found that public compliance had an indirect conditional impact on the efficacy of social distancing policy. Therefore, we argued that public health policymaking against the pandemic is supposedto sufficiently integrate the public perspective. With respect to the statistical analysis, we used the variable of restrictions on gatherings to represent social distancing policy, and acquired several findings. Firstly, in the cross-national section, population density is not associated with increasing confirmed cases, whereas the interaction of restriction on gatherings and population density achieves the expected outcome. This result highlights the importance of contextualizing social distancing. Secondly, in the section on the fixed effect speculation, restrictions on gatherings exerted a significantly influential effect on their own. Together with the interaction effect of public compliance and a restriction on gatherings, they captured a greater explanatory power for the policy efficacy on containing the virus spread. Thirdly, the efficacy of restrictions on gatherings with different magnitude varied with time, which indicated that the policy duration should also be taken into account, especially when the restriction practices depend greatly on the public. Based on these findings, we generalized an argument: public compliance played a conditional role in social distancing efficacy by contextualizing with policy magnitude and policy duration. Earlier research found that the effectiveness of types of gathering bans was high but limited to the greatest magnitude [63]. Our findings added that even gathering restrictions with lighter magnitudes captured effectiveness, and its impact could last for a certain time period.

Our findings coincided with the consensus that, in the absence of clear medical countermeasures, social contact reduction as part of the nonmedical interventions, is of emergent necessity in containing the disease at the initial stage of emergency responses. Even though public health experts have advocated social distancing approaches to policymakers, “how population behaviour patterns might most effectively be shifted remains one of the greatest uncertainties for research and policy” [65]. Our study provided an alternative solution to this problem. We suggested adjusting public policy formulation and implementation by taking public compliance into account at the problem-framing phase, which could bring about a more sustained containment picture of the pandemic. Some studies isolated the impact evaluation of public health policy with little consideration for the public perspective. To complement this, we initiated using population density as a proxy variable of public compliance’s role related to policy efficacy. The analysis showed that suitably restrictive measures performed sustainably better than the strictest ones, though stronger restrictions may bring greater immediate effects. Hence, all the results could contribute to the current debate of the necessity of restriction policies and the need to convince people within high-risk areas to comply. On the basis of this exploration, our future work will continue to attempt collective compliance measurement and its influence on social distancing policy by performing empirical research on actual compliance conditions.

Our investigation may provide insight into the possibility of coordinating public health policy at the country level globally. Introducing policy interventions with an appropriate magnitude as early as possible was suggested to decrease the spillover effects of cross-national, poorly coordinated responses [66]. Though COVID-19 spillover has been partially predicted, its worldwide consequences and social costs remain to be assessed [67,68]. In addition, we are aware of the uncertainties caused by collective compliance, and addressing them in the policy design phase could improve emergency preparedness to tackle future epidemics or even global health events. Hopefully, our research can raise policymakers’ awareness of appreciating the values and contributions of the general public to combat COVID-19, and also other emerging pandemics, and encourage them to realize that the strictest implementation of the hardest measures, despite being in emergent situations, owe the global public great thanks. COVID-19 has been regarded as a ‘common tragedy’ due to disobedient behavior options. While viewing this global phenomenon in our study, it can be understood as a global public enlightenment in arguing for the evidence-based policy design.

Last but not least, there are some limitations to our research that should be noted. Firstly, as the outbreaks of COVID-19 occurred at different time points in different countries and territories, the discontinuity between real-time spread and data reports may bias the estimation of timing effects globally. Secondly, the technical method has limitations. Concerning the pooling intercepts and errors, the effect of individual-based factors is connected with the variables [69]. This correlation can shape the distribution of error terms and the value of interception [70]. Furthermore, although this study can provide an initial exploration to evaluate social distancing policy interacting with public compliance in the global context, other influential variables remain to be identified and included in the model. For example, on an ethical basis, public health authorities’ credibility might affect the mitigating efficacy of social distancing policy [71]. Additionally, knowledge about new infectious diseases keeps being updated continuously, and uncertainty might underestimate the optimal impact of confinement policy [72,73]. It takes time to trace the virus’ origin and morbidity patterns, and a lack of evidence hinders further research into identifying evolving scenarios in heterogeneous social contexts [74]. Our study was a preliminary analysis without considering the heterogeneity of contexts. Further research can be progressed on how to test the conditions for later waves caused by COVID-19 variants. Furthermore, social distancing policy is a broad concept containing many options involving various places with different magnitudes. This paper selects gathering restrictions due to their more concise advantage, yet a much clearer categorical division is required to clarify the issue from a considerately complex perspective. For the above-mentioned reasons, further investigations on this topic using more specific regional subsamples or other time subsamples are needed to consider public compliance in the appropriateness of health policy design, which matters to the total containment efficacy as everyone counts, eventually.

## Figures and Tables

**Table 1:** Descriptive statistics of variables for analysis.

Variables	Data Sources	Observations	Mean	Std. Dev.	Min	Max
cases	ECDC	17,812	373.236	2006.523	0	48,529
gre	OxCGRT	17,812	-	-	0	1
days	OxCGRT	17,812	28.034	27.810	0	141
resg	OxCGRT	17,812	-	-	0	4
dnst	World Bank	17,812	237.710	780.456	0.136	7952.998

Note: The variable of “cases” represents the daily number of new COVID-19 cases. The variable of “gre” represents whether a country or territory takes restrictive measures on gatherings or not, 0 means the country or territory adopts no confinement policy, and 1 represents that the country or territory introduces certain restrictive measures. In this dataset, category 0 occupies 5788 observations, while, for 1 it involves 12,024 observations. The variable of “days” represents the cumulative duration that a country or territory undertakes a certain degree of gathering ban in the research period, and the specific coding information concerning the variable of ‘days’ is listed in the Supplementary Materials. The variable of “resg” represents the magnitude of restrictions on gatherings, which includes four ordinal scales: 1 signifies a restriction on gatherings of more than 1000 people, 2 stands for a restriction on gatherings of between 101 and 1000 people, 3 represents a restriction on gatherings of between 11 and 100 people, and 4 is the restriction on gatherings of no more than 10 people. In this study, we renamed these types of gathering bans as Level 1, Level 2, Level 3, and Level 4, respectively. The distribution of these levels is 3.00% (Level 1), 5.51% (Level 2), 32.77% (Level 3) and 58.72% (Level 4). The variable of “dnst” represents the population density, and its definition is people per square kilometers of land area.

**Table 2:** Random effect estimates of adopting restrictions on gatherings.

Dependent Variable: Cases	Model 1	Model 2	Model 3
Restriction on Gatherings (resg)	938.265 *** (29.874)		979.915 *** (31.282)
Population Density (dnst)		−0.035 (0.127)	0.073 (0.136)
Restriction on Gatherings × Population Density			−0.135 *** (0.030)
Constant	−393.345 *** (90.551)	297.389 *** (86.851)	−420.428 *** (95.387)
Observations	17,812	17,812	17,812
Log Likelihood	−156,363.800	−156,848.100	−156,357.600
Akaike Inf. Crit.	312,735.600	313,704.100	312,727.200
Bayesian Inf. Crit.	312,766.800	313,735.300	312,773.900

Note: Standard errors are in parentheses. Significance levels: *** *p* < 0.01.

**Table 3:** Fixed effect estimates of adopting a particular restriction level.

Dependent Variable: Cases	Model 1	Model 2	Model 3
Level 1	−436.224 (280.420)	16.019 (231.021)	−658.557 *** (246.059)
Level 2	−485.387 ** (208.981)	−472.733 ** (208.277)	−1147.309 *** (225.623)
Level 3	−403.168 ** (197.717)	−403.980 ** (197.840)	−1078.556 *** (221.733)
Level 4	383.896 * (205.047)	347.572 * (204.914)	−327.004 (217.733)
Population Density (dnst)	2.697 ** (1.371)	−1.147 *** (0.439)	
Level 2 × Population Density	−3.758 *** (1.302)		
Level 3 × Population Density	−3.773 *** (1.302)		
Level 4 × Population Density	−3.898 *** (1.301)		
Observations	12,024	12,024	12,024
R^2^	0.734	0.733	0.733
Adjusted R^2^	0.727	0.726	0.726
Residual Std. Error	1291.405 (df = 11,723)	1292.377 (df = 11,726)	1292.377 (df = 11,726)
F Statistic	107.291 *** (df = 301; 11,723)	108.139 *** (df = 298; 11,726)	108.139 *** (df = 298; 11,726)

Note: Standard errors are in parentheses. Parameters are estimated using the least squares dummy variable (LSDV) model, including country/territory and days (factored respectively). Significance levels: * *p* < 0.1; ** *p* < 0.05; *** *p* < 0.01.

**Table 4:** Fixed effect estimates of restriction efficacy over time.

Time Interval	Restriction on Gatherings Level:			
	Level 1	Level 2	Level 3	Level 4
3 days	−3.277	−3.375 *	−2.738 **	−4.738 **
	(2.011)	(1.855)	(1.301)	(2.052)
7 days	0.803	−4.646 ***	−3.612 ***	−3.267 **
	(1.666)	(1.359)	(1.225)	(1.337)
15 days	−1.227	−3.662 ***	−2.563 ***	−1.930 **
	(0.924)	(0.868)	(0.824)	(0.837)
30 days	−1.764 **	−2.454 ***	−2.308 ***	−1.785 ***
	(0.788)	(0.701)	(0.660)	(0.676)
40 days	−2.028 ***	−1.711 ***	−1.780 ***	−1.654 ***
	(0.770)	(0.663)	(0.619)	(0.637)
50 days	−2.262 ***	−1.582 **	−1.813 ***	−1.453 **
	(0.782)	(0.660)	(0.622)	(0.633)
60 days	−2.495 ***	−1.576 **	−1.945 ***	−1.269 **
	(0.791)	(0.658)	(0.623)	(0.630)

Note: Standard errors are in parentheses. The dependent variable (“cases”) has been transformed by logging in the equation, in which the interaction of restrictions on gatherings and population density is included. As the research concern moves on to the perspective of public policy, the figures above refer to the individual efficacy of the particular gathering ban over days. Significance levels: * *p* < 0.1; ** *p* < 0.05; *** *p* < 0.01.

## Data Availability

The paper combines three publicly archived datasets, including COVID-19 coronavirus data. Available online: https://data.europa.eu/euodp/en/data/dataset/covid-19-coronavirus-data (accessed on 15 June 2020), the Oxford COVID19 government response tracker (OxCGRT). Available online: https://covidtracker.bsg.ox.ac.uk/ (accessed on 27 December 2021), and Population density (the World Bank’s data). Available online: https://data.worldbank.org/indicator/EN.POP.DNST?view=chart (accessed on 27 December 2021). Under the data use policies, original datasets are available from the corresponding author upon reasonable request.

## Supplementary Materials

The following supporting information can be downloaded at: https://www.mdpi.com/article/10.3390/ijerph19074033/s1.
