# Supplementary material for: Public Compliance Matters in Evidence-Based Public Health Policy: Evidence from Evaluating Social Distancing in the First Wave of COVID-19
Source: Int J Environ Res Public Health. 2022 Mar 29;19(7):4033. doi: 10.3390/ijerph19074033 (PMC8997917; doi:10.3390/ijerph19074033)
Supplement: Supplementary file 1 [file ijerph-19-04033-s001.zip › ijerph-1551851-supplementary.pdf]

S1 – Model

To evaluate the mitigation efficacy of social distancing, we focus on the side of policy supply while assuming the compliance of policy is endogenous in all countries and territories at the demand side. Allowing for the evaluation at the same time, a simple statics model would be

$$E(\Pi_\rho) = \alpha + \beta_1\lambda_\nu\tau + \beta_2\delta_\nu + \beta_3\lambda_\nu\delta_\nu\tau + \epsilon \quad \text{(S1)}$$

Where Π_ρ refers to new cases infected in one geographical area at a certain time point, ρ denotes the geographical locations referring to countries and territories, λ_v stands for specific social distancing categorized by magnitude, τ denotes the time factor related to social distancing implementation, and δ_v represents the population density in a specific geographical area. The model setting focuses on the spread of COVID-2019 in a particular geographical coverage at a certain timing point. we observe that many countries take systematic response rather than just social distancing, which means that travel ban, border control and lock down can keep the growth of new cases in a less connected way.

Consider two types of social distancing measures, the loose one and the strict one:

$$\lambda 1 < \lambda 2 \quad \text{(S2)}$$

Assume two geographical areas with different population density, the small one and the big one:

$$\delta 1 < \delta 2 \quad \text{(S3)}$$

First differencing the model with the same time point:

$$E(\Pi_{\rho_1}) = \alpha_{\rho_1} + \beta_1\lambda_1\tau + \beta_2\delta_1\tau + \beta_3\lambda_1\delta_1\tau + \epsilon_{\rho_1} \quad \text{(S4)}$$

$$E(\Pi_{\rho_2}) = \alpha_{\rho_2} + \beta_4\lambda_2\tau + \beta_5\delta_2 + \beta_6\lambda_2\delta_2\tau + \epsilon_{\rho_2} \quad \text{(S5)}$$

Under full social distancing impact condition, irrelevant of the time point and geographical areas, $\beta_1 = \beta_4$, $\beta_2 = \beta_5$, $\beta_3 = \beta_6$[16]. Pooling intercepts and errors in the two models remains to be discussed later in the limitation section, simply for comparing the main parts,

$$E(\Pi_{\rho 2}) - E(\Pi_{\rho 1}) = (\beta_4 - \beta_1)\lambda_1\tau + (\beta_5 - \beta_2)\delta_2 + (\beta_6 - \beta_3)\lambda_2\delta_2\tau \quad \textit{(S6)}$$

Assuming that new daily cases keeps increasing, then $E(\Pi_{\rho 2}) > E(\Pi_{\rho 1})$, consequently $(\beta_4 - \beta_1)\lambda_1\tau + (\beta_5 - \beta_2)\delta_2 > -(\beta_6 - \beta_3)\lambda_2\delta_2\tau$.

Let $\sigma_1 = \beta_4 - \beta_1$, $\sigma 2 = \beta_5 - \beta_2$, $\sigma 3 = \beta_6 - \beta_3$, the comparing function turns to be $\sigma_1\lambda_1\tau + \sigma_2\delta_2 > -\sigma_3\lambda_2\delta_2\tau$, put the two sides into natural log, log $(\sigma_1\lambda_1\tau\sigma_2\delta_2)$ = log $(-\sigma_3\lambda_2\delta_2\tau)$, based on this result, $\sigma_1\lambda_1\tau\sigma_2\delta_2$

$=-\sigma_3\lambda_2\delta_2\tau$, so

$$\frac{\lambda_1}{\lambda_2} = -\frac{\sigma_3}{\sigma_1\sigma_2} \tag{S7}$$

As explained earlier, $\lambda_1 < \lambda_2$, thus $\sigma_3 + \sigma_1\sigma_2 > 0$. Generally there is a positive correlation between the infected number and population density, therefore $\sigma_2 > 0$, dividing by σ_2, then $\sigma_3/\sigma_2 > -\sigma_1$, returning the original parameter, the equation becomes $\beta_6 - \beta_3/\beta_5 - \beta_2 > \beta_1 - \beta_4$. If $\beta_5 - \beta_2 > 0$, then $\beta_6 - \beta_3 > (\beta_1 - \beta_4)(\beta_5 - \beta_2)$, following this, $\beta_6 - \beta_3 > 0|\beta_4 - \beta_1 > 0$, or $\beta_4 - \beta_1 > 0|\beta_6 - \beta_3 < 0$. These two cases present different pictures. One picture is that conditional on the positive coefficient of population density, both the social distancing and the correlation between social distancing and population density identically have positive effects on the reproduction of new cases, whereas the other picture is that both population density and social distancing have positive signs while the interaction between them can exert negative effect.

In the situation of negative correlation between population density and infected cases growth, $\beta_5 - \beta_2 < 0$, then $\beta_6 - \beta_3 < (\beta_1 - \beta_4)(\beta_5 - \beta_2)$. There are also two pictures, one is $\beta_6 - \beta_3 < 0|\beta_4 - \beta_1 > 0$, and another possible picture is that $\beta_4 - \beta_1 < 0|\beta_6 - \beta_3 > 0$, with the latter holds under certain conditions. For the first picture, when the direction of social distancing sign is counter to that of the interaction between social distancing and population density, the effect of their interaction is negative. However, as regards the second picture, it is occasionally holds that the population density has a different direction with that of the interaction term; the social distancing effect shows negative. To sum up, geographic factor consists of conditions that constraint the effect of social distancing and the interaction term composed of social distancing and population density.

Considering the period, under partial social distancing impact condition, parameters have unequal relations. Let $\rho_{\delta_\nu}^{\lambda_\nu}$ denote the match pair of social distancing restriction and population density, there are four scenarios:

$\rho_{\delta_1}^{\gamma_1}$ signifies less social distancing restriction in lower population density

$\rho_{\delta_2}^{\gamma_1}$ signifies less social distancing restriction in higher population density

$\rho_{\delta_1}^{\gamma_2}$ signifies more social distancing restriction in lower population density

$\rho_{\delta_2}^{\gamma_2}$ signifies more social distancing restriction in higher population density

Making pairs for the comparison, the first pair is the different social distancing restriction and the

different population density: $\rho_{\delta_1}^{\gamma_1}$ and $\rho_{\delta_2}^{\gamma_2}$, $\rho_{\delta_2}^{\gamma_1}$ and $\rho_{\delta_1}^{\gamma_2}$; The second pair is the different social distancing restriction and the same population density: $\rho_{\delta_1}^{\gamma_1}$ and $\rho_{\delta_1}^{\gamma_2}$, $\rho_{\delta_2}^{\gamma_1}$ and $\rho_{\delta_2}^{\gamma_2}$; The third pair contains the same social distancing restriction and the different population density: $\rho_{\delta_1}^{\gamma_1}$ and $\rho_{\delta_2}^{\gamma_1}$, $\rho_{\delta_1}^{\gamma_2}$ and $\rho_{\delta_2}^{\gamma_2}$. This paper focuses on policy impact evaluation. The first pair is beyond the coverage, the second pair actually considers time factor with an emphasis on time-series analysis, and the third pair stresses on the cross-sectional analysis.

Assume two time periods at which the two authorities in different geographical areas begin to implement social restriction measures:

$$\tau 1 < \tau 2 \tag{S8}$$

Adding the interval between the two time points:

$$\tau_i = \tau_2 - \tau_1 \tag{S9}$$

$$i \in (1,n)$$

Adding the difference of time point in differencing models, four dynamics models are generated:

$$E(\Pi_{\rho_1}) = \alpha_{\rho_1} + \beta_1\lambda_1\tau_1 + \beta_2\delta_1\tau_1 + \beta_3\lambda_1\delta_1\tau_1 + \epsilon_{\rho_1} \tag{S10}$$

$$E(\Pi_{\rho_1}) = \alpha_{\rho_1} + \beta_1\lambda_1\tau_2 + \beta_2\delta_1\tau_2 + \beta_3\lambda_1\delta_1\tau_2 + \epsilon_{\rho_1} \tag{S11}$$

$$E(\Pi_{\rho_2}) = \alpha_{\rho_2} + \beta_4\lambda_2\tau_1 + \beta_5\delta_2\tau_1 + \beta_6\lambda_2\delta_2\tau_1 + \epsilon_{\rho_1} \tag{S12}$$

$$E(\Pi_{\rho_2}) = \alpha_{\rho_2} + \beta_4\lambda_2\tau_2 + \beta_5\delta_2\tau_2 + \beta_6\lambda_2\delta_2\tau_2 + \epsilon_{\rho_2} \tag{S13}$$

Adopting the former practice, the equations can be transformed by replacing the time point with interval:

$$E(\Pi'_{\rho_1}) = \alpha'_{\rho_1} + \beta'_1\lambda_1\tau_i + \beta'_2\delta_1\tau_i + \beta'_3\lambda_1\delta_1\tau_i + \epsilon_{\rho_1} \tag{S14}$$

$$E(\Pi'_{\rho_2}) = \alpha'_{\rho_2} + \beta'_3\lambda_2\tau_i + \beta'_4\delta_2\tau_i + \beta'_5\lambda_2\delta_2\tau_i + \epsilon_{\rho_2} \tag{S15}$$

This manuscript aims to test alterations of β series over time in the section of fixed effect analysis.

S2 – Sample

Table S1. List of Geographical Areas in the Sample (Names)

Countries and Territories					
Aruba	France	Angola	Albania	Andorra	Denmark
Argentina	Afghanistan	Austria	Azerbaijan	Burundi	Belgium
Benin	Australia	Bangladesh	Bulgaria	Bahrain	Côte d'Ivoire
Belarus	Burkina Faso	Bermuda	Cameroon	Brazil	Barbados
Chile	Belize	Botswana	Bhutan	Canada	Switzerland
Brunei Darussalam	Central African Republic	Bosnia and Herzegovina	Trinidad and Tobago	Korea, Republic of	United Arab Emirates
Colombia	China	Costa Rica	Cuba	Cyprus	Czech Republic
Germany	Cape Verde	Dominica	Romania	Dominican Republic	Algeria
Ecuador	Djibouti	Spain	Estonia	Ethiopia	Finland
Fiji	Egypt	Gabon	United Kingdom	Georgia	Ghana
Guinea	Gambia	Greece	Greenland	Guatemala	Guam
Guyana	Honduras	Croatia	Haiti	Hungary	Indonesia
India	Ireland	Portugal	Iraq	Iceland	Israel
Italy	Jamaica	Jordan	Japan	Kazakhstan	Kenya
Kyrgyzstan	Cambodia	Saudi Arabia	Kuwait	Congo	Lebanon
Liberia	Puerto Rico	Sri Lanka	Lesotho	Lithuania	Luxembourg
Morocco	Poland	Madagascar	Mexico	Mali	Myanmar
Mongolia	Mozambique	Mauritania	Mauritius	Malawi	Malaysia
Namibia	Niger	Nigeria	Nicaragua	Netherlands	Norway
Nepal	New Zealand	Oman	Pakistan	Panama	Peru
Russian Federation	Papua New Guinea	Moldova, Republic of	Libyan Arab Jamahiriya	Iran, Islamic Republic of	Syrian Arab Republic
Palestinian Territory, Occupied	Bolivia, Plurinational State of	Lao People's Democratic Republic	Tanzania, United Republic of	Venezuela, Bolivarian Republic of	Congo, the Democratic Republic of the
Senegal	Singapore	Sierra Leone	El Salvador	San Marino	Somalia
Serbia	Suriname	Slovakia	Slovenia	Sweden	Swaziland
Seychelles	Paraguay	Chad	Thailand	Tajikistan	Timor-Leste
Qatar	Tunisia	Turkey	Philippines	Uganda	Ukraine
Uruguay	United States	Uzbekistan	Rwanda	Viet Nam	Yemen
South Africa	Zambia	Zimbabwe			

Table S2. List of Geographical Areas in the Sample (Codes)

Countries and Territories					
ABW	FRA	AGO	ALB	AND	ARE
ARG	AFG	AUT	AZE	BDI	BEL
BEN	AUS	BGD	BGR	BHR	BIH
BLR	BFA	BMU	BOL	BRA	BRB
BRN	BLZ	BWA	CAF	CAN	CHE
CHL	BTN	CIV	CMR	COD	COG
COL	CHN	CRI	CUB	CYP	CZE
DEU	CPV	DMA	DNK	DOM	DZA
ECU	DJI	ESP	EST	ETH	FIN
FJI	EGY	GAB	GBR	GEO	GHA
GIN	GMB	GRC	GRL	GTM	GUM
GUY	HND	HRV	HTI	HUN	IDN
IND	IRL	IRN	IRQ	ISL	ISR
ITA	JAM	JOR	JPN	KAZ	KEN
KGZ	KHM	KOR	KWT	LAO	LBN
LBR	LBY	LKA	LSO	LTU	LUX
MAR	MDA	MDG	MEX	MLI	MMR
MNG	MOZ	MRT	MUS	MWI	MYS
NAM	NER	NGA	NIC	NLD	NOR
NPL	NZL	OMN	PAK	PAN	PER
PHL	PNG	POL	PRI	PRT	PRY
PSE	QAT	ROU	RUS	RWA	SAU
SEN	SGP	SLE	SLV	SMR	SOM
SRB	SUR	SVK	SVN	SWE	SWZ
SYC	SYR	TCD	THA	TJK	TLS
TTO	TUN	TUR	TZA	UGA	UKR
URY	USA	UZB	VEN	VNM	YEM
ZAF	ZMB	ZWE			

Note : The website of ISO 3166-1 alpha-3 codes is as follows:

http://unstats.un.org/unsd/tradekb/Knowledgebase/50347/Country-Code.

S3 – Abbreviations in the Manuscript and supplementary

I. A List of Variables in the Manuscript

cases: The daily number of new COVID-19 cases.

gre: Whether a country or territory take restrictive measures on gathering or not, “0” means the country or territory takes no confinement policy, “1” signifies that the country or territory introduces certain restrictive measures. This variable is coded on the basis of the variable of “restrictions on gathering” in the Oxford COVID19 Government Response Tracker (OxCGRT) data. The original variable had five ordinal scales, covering “0”, “1”, “2”, “3”, “4”. In this article, we divided it into two variables, namely, “gre” and “resg”.

resg: As explained above, this variable represents the magnitude of restrictions on gatherings, which includes four ordinal scales: “1” signifies restriction on gatherings of more than 1,000 people, “2” stands for restriction on gatherings of between 101-1,000 people, “3” represents restriction on gatherings of between 11-100 people, “4” is the restriction on gatherings of no more than 10 people, and we rename these types of gathering ban by Level 1, Level 2, Level 3, and Level 4 accordingly.

Level 1: The restriction on gatherings of more than 1,000 people.
Level 2: The restriction on gatherings of between 101-1,000 people.
Level 3: The restriction on gatherings of between 11-100 people.
Level 4: The restriction on gatherings of no more than 10 people.

days: The cumulative duration of a country or territory undertook certain degree of gathering ban in the research period. Due to the variations of COVID-19 across countries and territories, policy interventions follow various patterns. Given that, the original time point is tailored for each country and territory respectively. With the mutating of virus, policy responses have experienced adjustment according to heterogeneous situations. Therefore, we take the policy as a whole circle to measure the accumulative

duration of particular measure. Moreover, this variable is designed based on the data of COVID-19 cases, which may confront discrepancies due to different updating time and time zone. In that regard, caution and awareness of limitations should be used in the statistical analysis.

3 days: Countries and territories have imposed a specific restriction on gathering in the period of three days.
7 days: Countries and territories have imposed a specific restriction on gathering in the period of seven days.
10 days: Countries and territories have imposed a specific restriction on gathering in the period of ten days.
15 days: Countries and territories have imposed a specific restriction on gathering in the period of fifteen days.
30 days: Countries and territories have imposed a specific restriction on gathering in the period of thirty days.
40 days: Countries and territories have imposed a specific restriction on gathering in the period of forty days.
50 days: Countries and territories have imposed a specific restriction on gathering in the period of fifty days.
60 days: Countries and territories have imposed a specific restriction on gathering in the period of sixty days.

dnst: The population density, which is defined by people per square kilometers of land area.

II. A List of Variables in the supplementary

geo_n: The amount of countries and territories.

ge_obs: The number of subject objections of relevant countries and territories in the whole sample.

geo_p: The percentage of geographical locations that accounts in studied sample.

ge_obs_p: The percentage of observations concerning application of particular restriction policy.

Deaths: The daily death number of COVID-19 in the studied period.

S4 – The Variant Duration of Restrictions on Gatherings

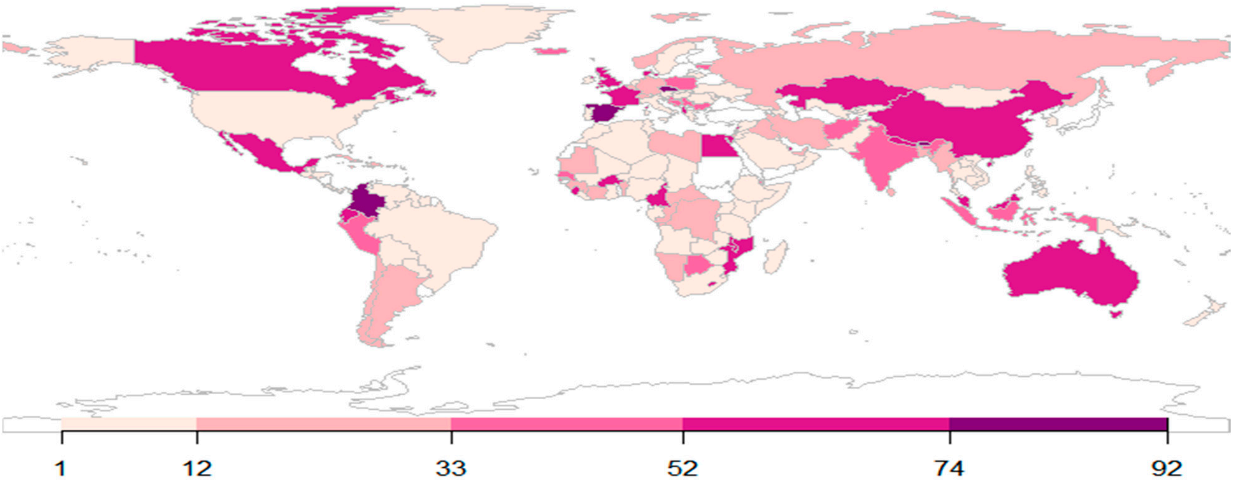

Note: The global map is self-made using R. The legend shows the period that restrictions on gatherings are exposed across countries and territories.

Data source: The initial data set is obtained from the Oxford Covid-19 Government Response Tracker (OxCGRT), and the variable of "days" is coded by the authors.

Figure S1. Aggregated Duration of the Restrictions on Gatherings in the First Wave of COVID-19

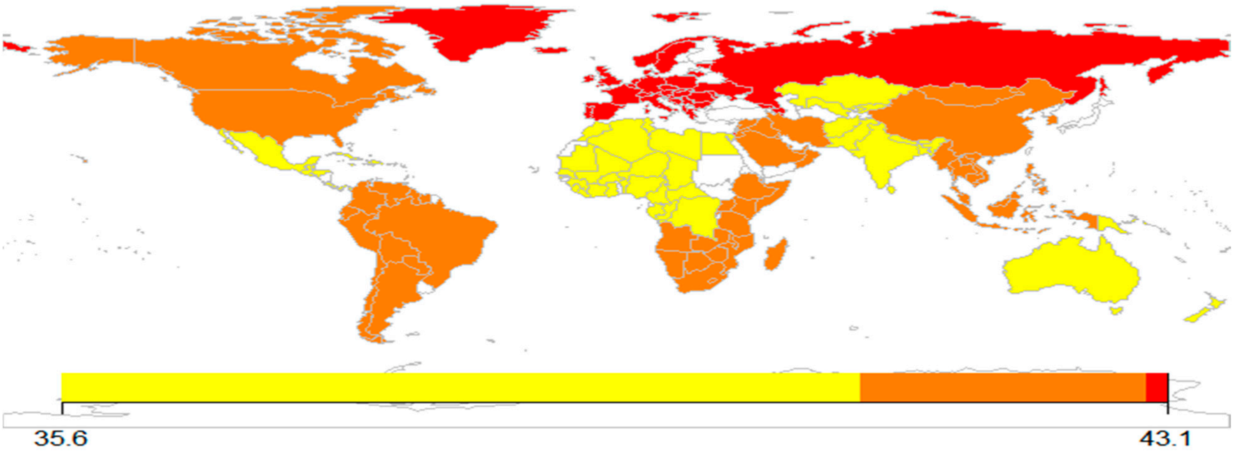

Note: The global map is self-made using R. We subset the data by 'cases' greater than 0 and there is presence of gathering ban in the studied period.

Data source: The initial data set is obtained from the Oxford Covid-19 Government Response Tracker (OxCGRT), and the variable of "resg" comes from the data set matched and re-coded by the authors.

Figure S2. Duration of Countries and Territories' Adopting Restrictions on Gatherings

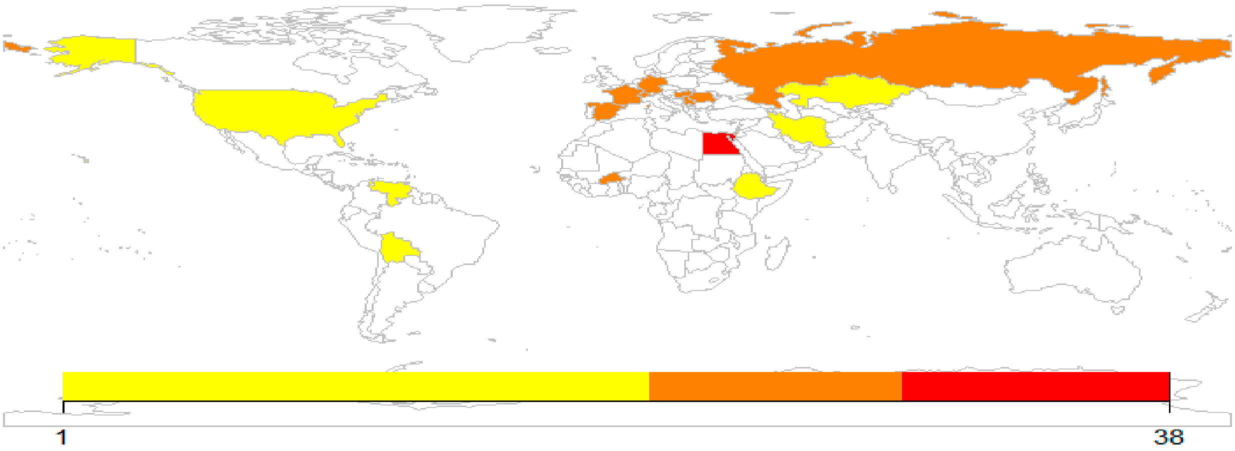

Note: The global map is self-made using R. We subset the data by 'cases' greater than 0 and "resg" equal to 1.

Data source: The initial data set is obtained from the Oxford Covid-19 Government Response Tracker (OxCGRT), and the variable of "resg" is generated from the data set matched and re-coded by the authors.

Figure S3. Duration of Introducing the Restriction on Gatherings of more than 1,000 people (Level 1)

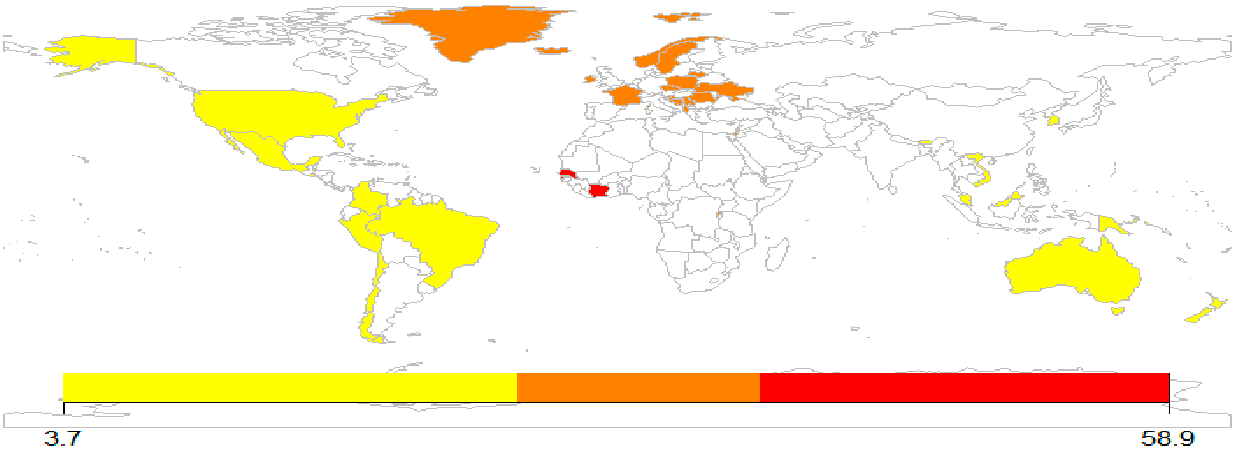

Note: The global map is self-made using R. We subset the data by 'cases' greater than 0 and "resg" equal to 2.

Data source: The initial variable of restrictions on gatherings is obtained from the Oxford Covid-19 Government Response Tracker (OxCGRT), and the variable of "resg" comes from the data set matched and re-coded by the authors.

Figure S4. Duration of Introducing the Restriction on Gatherings of between 101-1,000 people (Level 2)

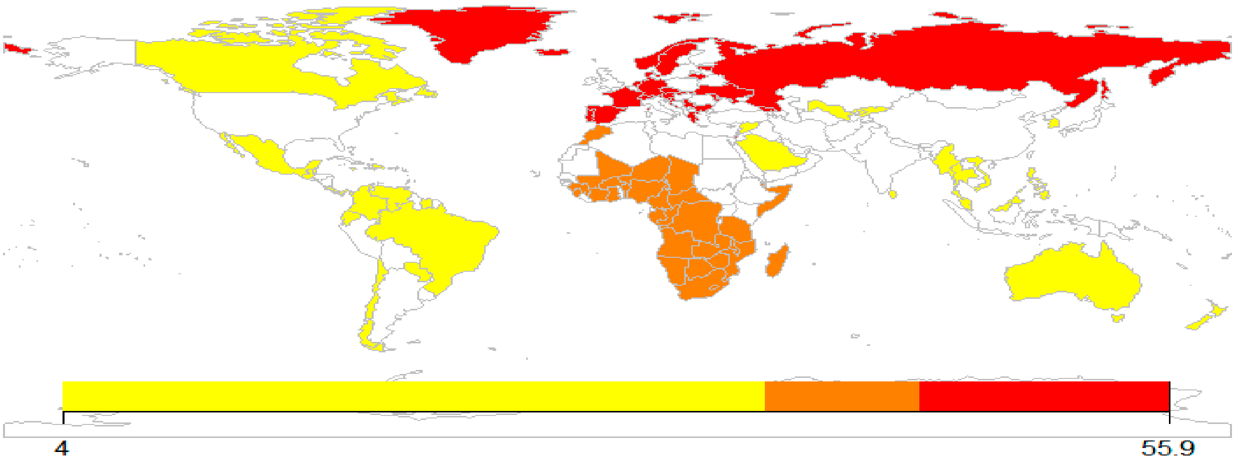

Note: The global map is self-made using R. We subset the data by cases' greater than 0 and "resg" equal to 3.

Data source: The initial variable of restrictions on gatherings is obtained from the Oxford Covid-19 Government Response Tracker (OxCGRT), and the variable of "resg" is from the data set matched and re-coded by the authors.

Figure S5. Duration of Introducing the Restriction on Gatherings of between 11-100 people (Level 3)

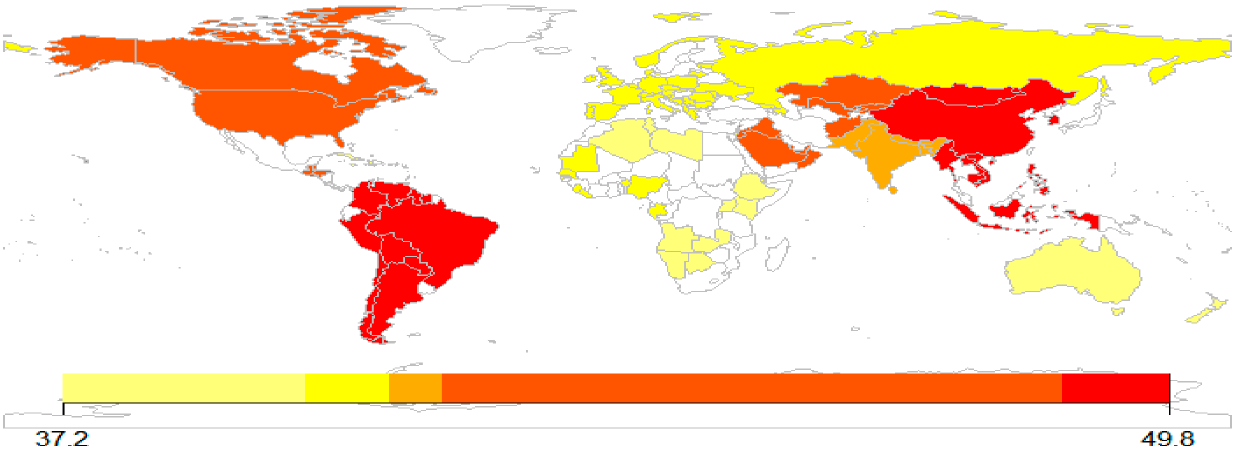

Note: The global map is self-made using R. We subset the data by cases' greater than 0 and "resg" equal to 4..

Data source: The initial variable of restrictions on gatherings is obtained from the Oxford Covid-19 Government Response Tracker (OxCGRT), and the variable of "resg" is from the data set matched and re-coded by the authors.

Figure S6. Duration of Introducing the Restriction on Gatherings of no more than 10 people (Level 4)

S5 – Distribution and Description of Restrictions on Gathering Types

Table S3. Distribution of Gathering Restriction Levels

Restriction Level	geo_n	ge_obs	geo_p (%)	ge_obs_p (%)
0	10	949	6.06	5.33
3	10	695	6.06	3.90
4	6	481	3.64	2.70
0, 1	3	400	1.82	2.25
0, 3	20	1,946	12.12	10.93
0, 4	33	3,893	20.00	21.86
1, 4	2	167	1.21	0.94
2, 3	2	156	1.21	0.88
2, 4	4	325	2.42	1.82
3, 4	10	777	6.06	4.36
0, 1, 3	1	81	0.61	0.45
0, 1, 4	1	80	0.61	0.45
0, 2, 3	5	706	3.03	3.96
0, 2, 4	6	648	3.64	3.64
0, 3, 4	25	2,878	15.15	16.16
0, 1, 2, 4	2	314	1.21	1.76
0, 1, 3, 4	4	629	2.42	3.53
0, 2, 3, 4	18	2,297	10.91	12.90
0, 1, 2, 3, 4	3	390	1.82	2.19

Note: The initial variable of restrictions on gatherings is obtained from the Oxford Covid-19 Government Response Tracker (OxCGRT), and the information above is collected by the authors.

Table S2 documented the process of taking different patterns of policy response. As is shown above, more than 155 countries and territories have adopted social gathering in the first wave of COVID-19. Imposing restriction on gathering in various way turned to be a global phenomenon. 50.30% of the studied geographical areas adjust restriction scales. In selecting the basic line, 7.88% of countries and territories take restrictions on gatherings of more than 1,000 people as starting point, 21% of geographical entities take restriction on gatherings between 101-1,000 people as policy foundation, of the rest, 21.21% of locations put restrictions on gatherings between 11 and 100 people in the first rank. Being contrary to restriction adjustments, 23.64% percent of geographical areas take the strictest measure with a ban on gatherings of no more than 10 people from the beginning. However, 6.06% of locations impose no gathering restrictions.

S6 – Other Tables

Table S4. Random Effect Estimate of Adopting Restrictions on Gathering

Dependent Variable: cases	Model: *Include Continent and Time*
Restriction on Gatherings	1,003.552**(211.945)
Population Density	0.057(0.136)
Restriction on Gatherings * Population Density	-0.138***(0.030)
Constant	-423.096(259.223)
Observations	17,812
Log Likelihood	-156,314.600
Akaike Inf. Crit.	312,645.200
Bayesian Inf. Crit.	312,707.500

Note: Standard errors are in parenthesis.

Significance levels: * p<0.1; ** p<0.05;*** p<0.01.

Table S5. Random Effect Estimate of Restriction on Gathering by Deaths

	Dependent Variable: Deaths
Restriction on Gatherings	64.473*** (12.180)
Population Density	0.005 (0.008)
Restriction on Gatherings * Population Density	−0.011*** (0.002)
Constant	−30.540* (15.638)
Observations	17,812
Log Likelihood	-109,089.000
Akaike Inf. Crit.	218,194.000
Bayesian Inf. Crit.	218,256.300

Note: Standard errors are in parenthesis.

Significance levels: * p<0.1; ** p<0.05;*** p<0.01.

Table S6. Random Effect Estimate of Adopting Particular Restriction on Gathering

	Dependent Variable: cases	
	Model 1	Model 2
Level 1	623.209*** (188.206)	752.023*** (275.627)
Level 2	366.131*** (76.154)	448.499** (215.684)
Level 3	460.584*** (45.952)	472.900** (206.876)
Level 4	1, 271.758*** (34.604)	1, 291.592*** (204.720)
Population Density	0.051 (0.136)	0.050 (0.136)
Level1 * Population Density	0.748 (1.412)	1.211 (1.408)
Level2 * Population Density	−0.073 (0.057)	−0.049 (0.057)
Level3 * Population Density	−0.073 (0.059)	−0.064 (0.059)
Level4 * Population Density	−0.186*** (0.033)	−0.196*** (0.032)
Constant	−368.827***(95.082)	−370.885 (227.677)
Observations	17,812	17,812
Log Likelihood	-156,155.900	-156,124.200
Akaike Inf. Crit.	312,335.900	312,276.500
Bayesian Inf. Crit.	312,429.300	312,385.500

Note: Standard errors are in parenthesis.

Significance levels: * p<0.1; ** p<0.05;*** p<0.01.

Table S7. Fixed Effect Estimate of Restrictions on Gatherings Within 3 Days, 7 Days and 15 Days

	Dependent Variable: Log (cases)		
	3 days	7 days	15 days
Level 1	- 3.277 (2.011)	0.803 (1.666)	- 1.227 (0.924)
Level 2	- 3.375* (1.855)	- 4.646***(1.359)	- 3.662***(0.868)
Level 3	- 2.738** (1.301)	- 3.612***(1.225)	- 2.563***(0.824)
Level 4	- 4.738** (2.052)	- 3.267**(1.337)	- 1.930**(0.837)
Log (Population Density)	1.194*** (0.364)	- 0.503 (0.394)	0.051 (0.177)
Level 2 * Log (Population Density)		1.538*** (0.361)	0.695*** (0.125)
Level 3 * Log (Population Density)		1.309*** (0.336)	0.456*** (0.111)
Level 4 * Log (Population Density)		1.194*** (0.327)	0.335*** (0.105)
Observations	308	758	1,693
R^2	0.978	0.969	0.967
Adjusted R^2	0.961	0.962	0.964
Residual Std. Error	0.626 (df = 173)	0.658 (df = 606)	0.712 (df = 1,521)
F Statistic	56.961*** (df = 135; 173)	126.664*** (df = 152; 606)	262.197*** (df = 172; 1,521)

Note: Standard errors are in parenthesis.

Significance levels: * p<0.1; ** p<0.05;*** p<0.01.

Table S8. Fixed Effect Estimate of Restrictions on Gatherings Within 30 Days, 40 Days, 50 Days and 60 Days

	Dependent Variable: Log (cases)			
	30 days	40 days	50 days	60 days
Level 1	- 1.764** (0.788)	- 2.028*** (0.770)	- 2.262*** (0.782)	- 2.495*** (0.791)
Level 2	- 2.454*** (0.701)	- 1.711*** (0.663)	- 1.582** (0.660)	- 1.576** (0.658)
Level 3	- 2.308*** (0.660)	- 1.780** * (0.619)	- 1.813*** (0.622)	- 1.945*** (0.623)
Level 4	- 1.785*** (0.676)	- 1.654*** (0.637)	- 1.453** (0.633)	- 1.269** (0.630)
Log (Population Density)	0.018 (0.152)	0.069 (0.152)	0.172 (0.157)	0.276* (0.161)
Level 2 * Log (Population Density)	0.322*** (0.106)	0.098 (0.109)	-0.029 (0.112)	- 0.115 (0.116)
Level 3 * Log (Population Density)	0.361*** (0.100)	0.152 (0.104)	0.047 (0.108)	- 0.048 (0.112)
Level 4 * Log (Population Density)	0.271*** (0.093)	0.183*(0.097)	0.050 (0.102)	- 0.081 (0.106)
Observations	3,527	4,696	5,833	6,975
R^2	0.962	0.958	0.953	0.950
Adjusted R^2	0.960	0.956	0.952	0.948
Residual Std. Error	0.825 (df = 3,338)	0.898 (df = 4,497)	0.963 (df = 5,624)	1.017 (df = 6,755)

F Statistic	450.774*** (df = 189; 3,338)	515.162*** (df = 199; 4,497)	551.766*** (df = 209; 5,624)	580.128*** (df = 220; 6,755)

Note: Standard errors are in parenthesis.

Significance levels: * p<0.1; ** p<0.05;*** p<0.01.

Table S9. Multinomial Regression Result on Social Distancing Policy Efficacy

Predictors	Odds Ratios	CI	P	Response
(Intercept)	0.07	0.07- 0.07	< 0.001	1
cases	1.00	1.00 - 1.00	< 0.001	1
Population Density	1.00	1.00 - 1.00	< 0.001	1
cases * Population Density	1.00	1.00 - 1.00	< 0.001	1
(Intercept)	0.11	0.11 - 0.11	< 0.001	2
cases	1.00	1.00 - 1.00	0.960	2
Population Density	1.00	1.00 - 1.00	0.171	2
cases * Population Density	1.00	1.00 - 1.00	< 0.001	2
(Intercept)	0.76	0.76 - 0.76	< 0.001	3
cases	1.00	1.00 - 1.00	0.316	3
Population Density	1.00	1.00 - 1.00	< 0.001	3
cases * Population Density	1.00	1.00 - 1.00	< 0.001	3
(Intercept)	1.02	1.02 - 1.02	< 0.001	4
cases	1.00	1.00 - 1.00	< 0.001	4
Population Density	1.00	1.00 - 1.00	< 0.001	4
cases * Population Density	1.00	1.00 - 1.00	< 0.001	4
Observations		17,812		
R^2 Nagelkerke		0.111		
log-Likelihood		-21,612.407		

Note: The social distancing policy is measured by the restrictions on gatherings.

Table S10. Estimate of Restriction on Gathering Efficacy in European Region

	Dependent Variable: Log (cases)	
	Least-squares Dummy Variables Model	*Linear Mixed-effects Model*
Level 1	36.515*** (2.365)	- 0.860 (1.610)
Level 2	38.834*** (2.245)	1.688 (1.475)
Level 3	39.321*** (2.263)	2.136 (1.438)
Level 4	42.031*** (2.243)	4.825*** (1.439)
Log (Population Density)	- 7.736*** (0.522)	0.968*** (0.344)
Level 2 * Log (Population Density)	- 0.406** (0.186)	- 0.459** (0.185)
Level3 * Log (Population Density)	- 0.596*** (0.168)	- 0.643*** (0.168)
Level 4 * Log (Population Density)	- 0.953*** (0.154)	- 0.972*** (0.154)
Observations	2,996	2,996
R^2	0.966	0.294
Adjusted R^2	0.964	0.294
Log Likelihood		-4,262.392
Akaike Inf. Crit.		8,546.784

Note: Standard errors are in parenthesis.

Significance levels: * $p<0.1$; ** $p<0.05$;*** $p<0.01$.
